# Acute effects of fruit flesh of two Saudi date varieties differing in phenolic content and compared with a sugar- and fibre-matched placebo on mood and cognition in healthy young volunteers: a randomised, double-blind, crossover study

**DOI:** 10.3389/fnut.2026.1733218

**Published:** 2026-07-22

**Authors:** Duaa Altuwairki, Anthony W. Watson, Helen J. Brooker, Kirsten Brandt

**Affiliations:** 1 Department of Nutrition and Food Science, Faculty of Human Sciences and Design, King Abdul-Aziz University, Jeddah, Saudi Arabia; 2 Human Nutrition and Exercise Research Centre, Population Health Sciences Institute, Newcastle University, Newcastle Upon-Tyne, United Kingdom; 3 School of Biomedical, Nutritional and Sports Sciences, Faculty of Medicine, Newcastle University, Newcastle Upon-Tyne, United Kingdom; 4 Medical School, University of Exeter, Exeter, United Kingdom; 5 Ecog Pro Ltd., Bristol, United Kingdom

**Keywords:** cognitive, dates, mood, performance, phenolics

## Abstract

**Purpose:**

This paper aimed to investigate the acute effect of crisp mature fruit flesh of two varieties of Saudi dates (Barhi and Khassab), containing 184 and 424 mg Gallic Acid Equivalents (GAE) of phenolic acids per portion, respectively, on cognitive performance, mood, and blood glucose concentration.

**Methods:**

The study employed a randomised, double-blind, placebo-controlled, crossover design with 35 healthy young participants aged 18–35 years, with a one-week washout period between visits. Computerised tests for attention and episodic memory were utilised to assess cognitive function at baseline and 45, 90 and 135 min post-consumption. Freeze-dried powder corresponding to 115 g fresh weight of pitted fruit was served in 150 g of yoghurt as the vehicle and placebo. All three treatments were matched regarding fibre and sugars.

**Results:**

For the individual task outcomes, after a Bonferroni correction (*α* = 0.0017), the only significant difference observed was a longer Digit Vigilance reaction time after consumption of Barhi (*p* = 0.001), indicating slower performance compared to Khassab, while Khassab did not differ from placebo. None of the cognitive indices reached significance (*α* = 0.0063). Regarding mood indices with α = 0.0167, consumption of Khassab significantly increased alertness (*p* = 0.005) and reduced calmness (*p* = 0.001), with no effect of Barhi (*p* > 0.4 for both outcomes). No significant changes in blood glucose levels were detected.

**Conclusion:**

Although a cautious statistical approach was employed, Khassab and Barhi date cultivars each showed different possible effects on specific areas of cognitive function in “young and healthy” participants, justifying future studies to assess replicability. The effects were not correlated with phenolic acid dose and did not represent general enhancement or deterioration of cognitive performance.

## Introduction

The date palm (*Phoenix dactylifera* L.) serves as a key source of nutrition and economic importance as a staple food in the diet of the Middle East, where it was originally domesticated (1, 2). Dates are consumed in three different edible maturation stages: Khalal or Bisr (crisp mature, 50% moisture), Rutab (soft mature, 30–35% moisture), and Tamr (dry mature, 10–30% moisture) (3).

Flavonoids and phenolic acids are the main phenolic compounds in date fruit. The two main subclasses of phenolic acids in dates are hydroxycinnamic acids and hydroxybenzoic acids (4, 5). The hydroxycinnamic acids include ferulic, p-coumaric, sinapic and caffeic acids, and the hydroxybenzoic acids include p-hydroxybenzoic, protocatechuic, vanillic, and syringic acids (6); however, factors like: soil environments, agronomic procedures and maturation stage can affect the contents of ferulic, sinapic acids and some specific cinnamic acid derivatives may vary between cultivars (7).

The primary focus of this study, which examines the outcomes on mood and cognitive functions of a date consumption treatment, is based on animal studies on the effect of dates on reversing cognitive impairment in controlled animal experiments employing ischaemic stroke models (8–13), in Alzheimer’s models (14–16) and on a substance-induced brain damage model (17). The authors of these animal studies suggested mechanisms of action of date phenolic compounds that enhanced memory to include anti-inflammatory and antioxidant responses, as well as improvements in neural signalling. Specifically, in studies with a transgenic mouse model of Alzheimer’s disease (14, 15, 18), the mice were given a diet containing either 2% or 4% date for 14 weeks and showed significant dose-dependent improvements of cognitive functioning, such as motor functions and spatial memory. The average daily consumption of whole fresh date fruit in KSA and UAE is 115 g (19) providing 324 calories, so the average date intake corresponds to 16% of the population’s overall calorie intake of ~2,000 kcal/day (20) in these countries. The present study’s treatment dose of 115 g fresh date fruit flesh was provided no more than once per week, thus providing approx. 2% of calories during the non-placebo weeks of the trial. Assuming that the energy density of the date material used in the rodent studies was similar to that of the mouse food it was incorporated into, our dose was therefore directly comparable with the quantities employed in the animal research literature, even though both rodent and human experimental doses were substantially lower than the average intake in a relevant population. In contrast, the body surface area (BSA) method for translating drug doses across species (21) could not be implemented for this comparison, since the daily quantities consumed of the mouse diet was not specified.

However, although the mitigation of symptoms of induced neurodegenerative diseases in these experimental animals were mainly attributed to the phenolic content of dates, there was a lack of details regarding the preparation of date-containing treatments utilised in these studies, resulting in challenges to compare the studies. In addition, other influences, such as locations, stages of fruit ripening and differing cultivars on phenolic content, increases the difficulty in determining the precise impact of date phytochemical compositions on the alleged neuroprotective actions (22, 23); making the association between the phenolic content of the date consumption and their neuroprotective actions inconclusive.

To the best of the authors’ knowledge, no peer-reviewed, placebo-controlled human studies have investigated the acute or chronic effects of the administration of date fruit on cognitive function and mood. Only one publication, a 2019 study by Abdullah et al. (24) investigated effects of the daily consumption of seven Ajwah date fruits for 6 weeks on cognitive processing. It demonstrated a significant enhancement of reaction time of the Stroop, 1-back and 2-back working memory tests and the tension subscale of the profile of mood scale (POMS) outcomes (24). However, these results are highly uncertain, since the study contained no control or placebo group; this also challenges the attribution of such results to the phenolic content, sugar, or other date constituents.

Evidence from studies on phenol-rich foods other than dates suggests that cognitive and mood-related effects may be partly attributed to their phenolic constituents, including flavonoids and phenolic acids. For example, cocoa flavanols have been associated with improvements in cognitive performance, sustained attention and mental fatigue in some acute and chronic interventions (25–27), while berry-based interventions, including grape, blueberry and blackcurrant products, have reported improvements in memory, attention or response speed (28–32). Importantly, berries contain both anthocyanins and phenolic acids (33), and one grape juice intervention specifically reported a polyphenol profile that included phenolic acids alongside anthocyanins and procyanidins (29). Therefore, given that date fruits are also reported to contain phenolic compounds, including phenolic acids (33), and in light of the wider evidence linking dietary polyphenols with cognitive outcomes (34), it was scientifically relevant to test whether the phenolic component of dates may contribute to effects on cognitive function and mood. Therefore, the present trial was designed to directly test this hypothesis in relation to dates, rather than assuming that the phenolic content alone explains any potential effects.

Therefore, this current study aimed to explore the acute effect of date-containing treatments differing in phenolic content, while matched for sugar and fibre, on cognitive function and mood. To allow placebo control, the freeze-dried powder form was chosen for two commercially available date cultivars in the Kingdom of Saudi Arabia (KSA). The two date cultivars were selected to differ in their phenolic acid content; therefore, the timing of the cognitive assessments was aligned with the maximum concentration Cmax of the phenolic acid levels; which is between 45- and 135-min post-consumption (35, 36). Yoghurt was chosen as vehicle and placebo due to its texture and taste being suitable to mask the presence of the date powder in the ‘active’ treatments, and colour and flavour masked in all three treatments.

## Methods

### Study design

The study followed a double-blind, counterbalanced, placebo-controlled, within-subjects design.

The trial was registered with ClinicalTrials.gov on 17 November 2017 (Identifier: NCT03350100; URL: https://clinicaltrials.gov/ct2/show/NCT03350100). All procedures involving human participants were reviewed and approved by Newcastle University’s Faculty of Medical Science ethics committee and conducted in accordance with the ethical standards of the 1964 Declaration of Helsinki and its subsequent amendments or comparable ethical principles.

### Participants

Through advertisement via opportunity sampling from Newcastle upon Tyne, UK, a total of 36 healthy young participants aged (18 to 35) were recruited, and each was given a £20 voucher as appreciation for their participation. The required number of participants was estimated using a power calculation based on a study that examined the acute effects of phenolic-rich blackcurrant treatments on mood and cognitive performance in healthy young adults and reported improvement in the accuracy of the Rapid Visual Information Processing (RVIP) task (32). A sample size of 34 participants was required to detect an actual effect between treatment conditions with 90% power, as calculated using G Power statistical software (version 3.0). The trial was conducted between October 2017 and March 2018. Prior to enrolment, each participant attended a 120-min screening and tasks familiarisation visit, which involved signing the consent and reporting themselves to be healthy, not consuming any over-the-counter medication or supplements, not pregnant, have no food allergies or sensitivities to the treatment constituents, being non-tobacco consumer, have a body mass index below 35 kg/m2 and not lower than 18 kg/m2. Descriptive statistic of the participant’s characteristics can be found in Table 1.

**Table 1:** Descriptive statistics of the participant characteristics (n = 35; 23 females and 12 males).

Measured characteristics	Min	Max	Mean	SD
Age	18	35	24	4.5
Height (cm)	145	191	168	11
Weight (kg)	43.1	98.3	67.5	14.5
BMI	18.2	31.0	24.2	3.5

### Study treatments

**Figure 1:**
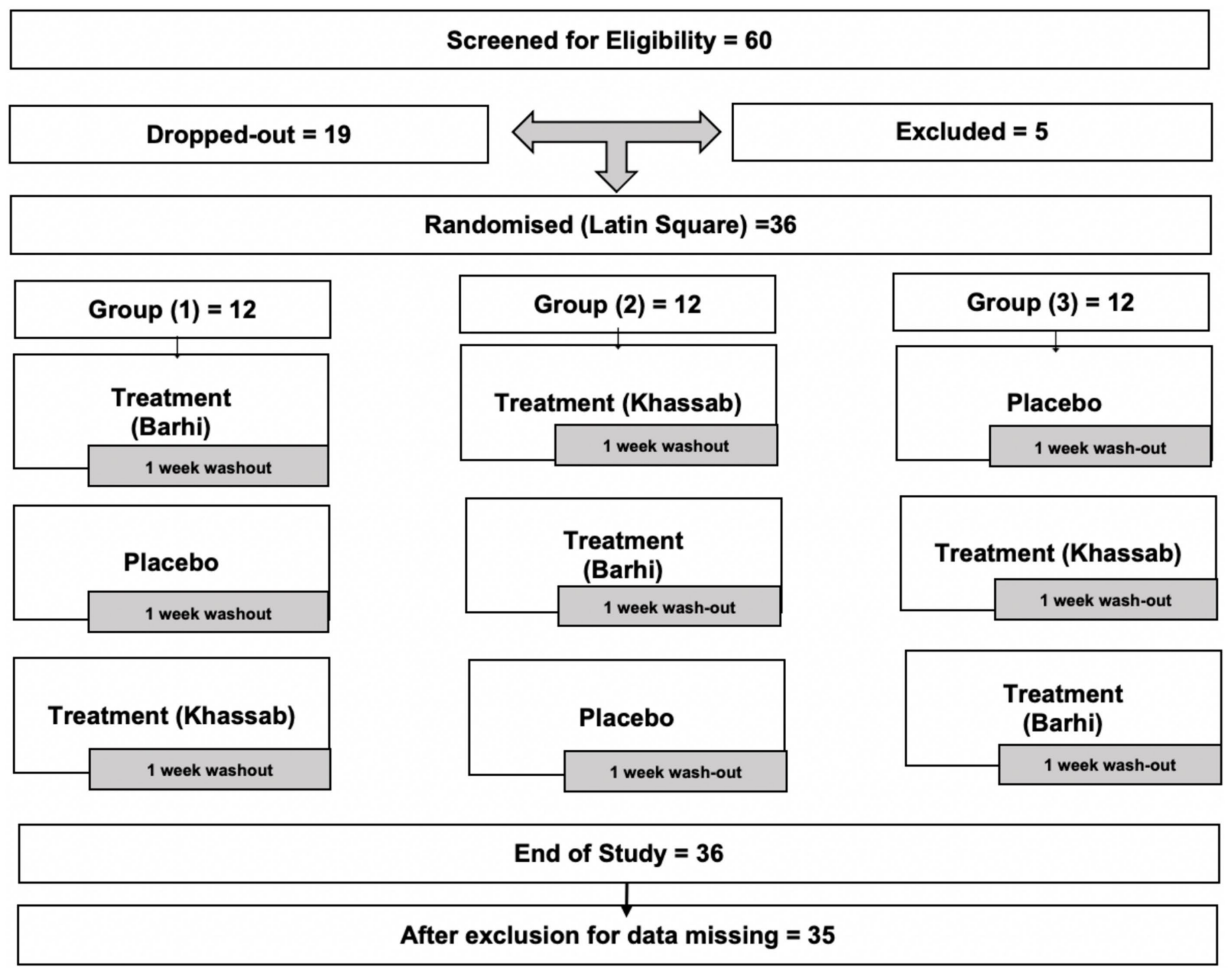
Overview of the study process and management of participants.

Participants were allocated to a treatment order by random assignment to a counterbalanced (Williams Latin Square) order, with a minimum one-week washout period between visits as shown in Figure 1. To ensure blinding, the treatments were prepared by an independent third party who had no other involvement in the conduct or analysis of the trial.

The study examined three treatments: Barhi, Khassab, and a placebo. The date fruits used were Barhi and Khassab cultivars in the Bisir maturation stage. The dates were freeze-dried by a commercial freeze-dry provider, European Freeze Dry Ltd. (Preston, United Kingdom).

The experimental dose of date in each date-containing treatment was 115 g/day; this was obtained with 48 g of Barhi and 34.5 g of Khassab in freeze-dried powder with 184 mg GAE/115 g FW and 424 mg GAE/115 g FW, respectively, as determined using the HPLC method as described in Alarcón-Flores et al. (37). The Barhi powder contained naturally occurring 16.78 grams of glucose and 15.84 grams of fructose without additional sugars. The Khassab powder contained 11.95 grams of glucose and 9.48 grams of fructose; thus, 4.84 grams of glucose and 6.36 grams of fructose were added. The placebo treatment comprised a minimal phenolic content of 16 mg per 150 grams and no natural sugars, prompting the addition of 16.78 grams of glucose and 15.84 grams of fructose for consistency. Similarly, the contents of insoluble and soluble fibre were equalised regarding both nutritional value and texture (blinding) by ‘topping up’ with purified fibre preparations to match the date powder with the highest natural content, see Table 2. All treatments were prepared in 0% fat yoghurt as the carrier matrix, with equal amounts of strawberry flavour and red + orange food colouring added to mask the flavour and colour of the date powder (Table 2). The process of developing the study treatments and placebo is further elaborated in (38). This choice followed extensive treatment development using different delivery methods, including unsuccessful alternatives such as date juice and capsule formats (38), and was supported by the widespread consumption of yoghurt (39).

**Table 2:** The treatment ingredients.

Ingredients	Barhi treatment	Khassab treatment	Placebo
Total phenolic content (TPC) as determined using HPLC	184 (mg/115 g FW) GAE	424 (mg/115 g FW) GAE	16 (mg/150 g) GAE
Date powder	48 g	34.5 g	0
0% fat yoghurt	150 g	150 g	150 g
Naturally available glucose	16.78 g	11.85 g	0
Added glucose for matching	0	4.94 g	16.78 g
Naturally available Fructose	15.84 g	9.36 g	0
Added fructose for matching	0	6.48 g	15.84 g
Naturally available soluble fibre	1.06 g	1.02 g	0
Added soluble fibre for matching	0	0	1.04 g
Naturally available insoluble fibre	4.08 g	6.54 g	0
Added insoluble fibre for matching	2.46 g	0	6.54 g
Strawberry flavour	5 drops	5 drops	5 drops
Red food colouring	3 drops	3 drops	3 drops
Orange food colouring	2 drops	2 drops	2 drops

Evidence suggests that polyphenol–protein interactions in dairy matrices do not necessarily reduce polyphenol bioavailability following digestion (40).

### Cognitive assessment tool CogTrack

To assess cognition, a set of nine online cognitive tests based on the original CDR System is available in the CogTrack system. The selected tests were pattern separation, immediate word recall, reaction time, choice reaction time, digit vigilance, working memory, delayed word recall, word recognition, numeric working memory, and pattern separation. These cognitive tasks have previously demonstrated sensitivity, validity, and reliability in detecting cognitive changes enhanced by an energy drink (41) and blackcurrant juice (31).

### Indices of cognitive assessment core measures from the CogTrack tasks

**Figure 2:**
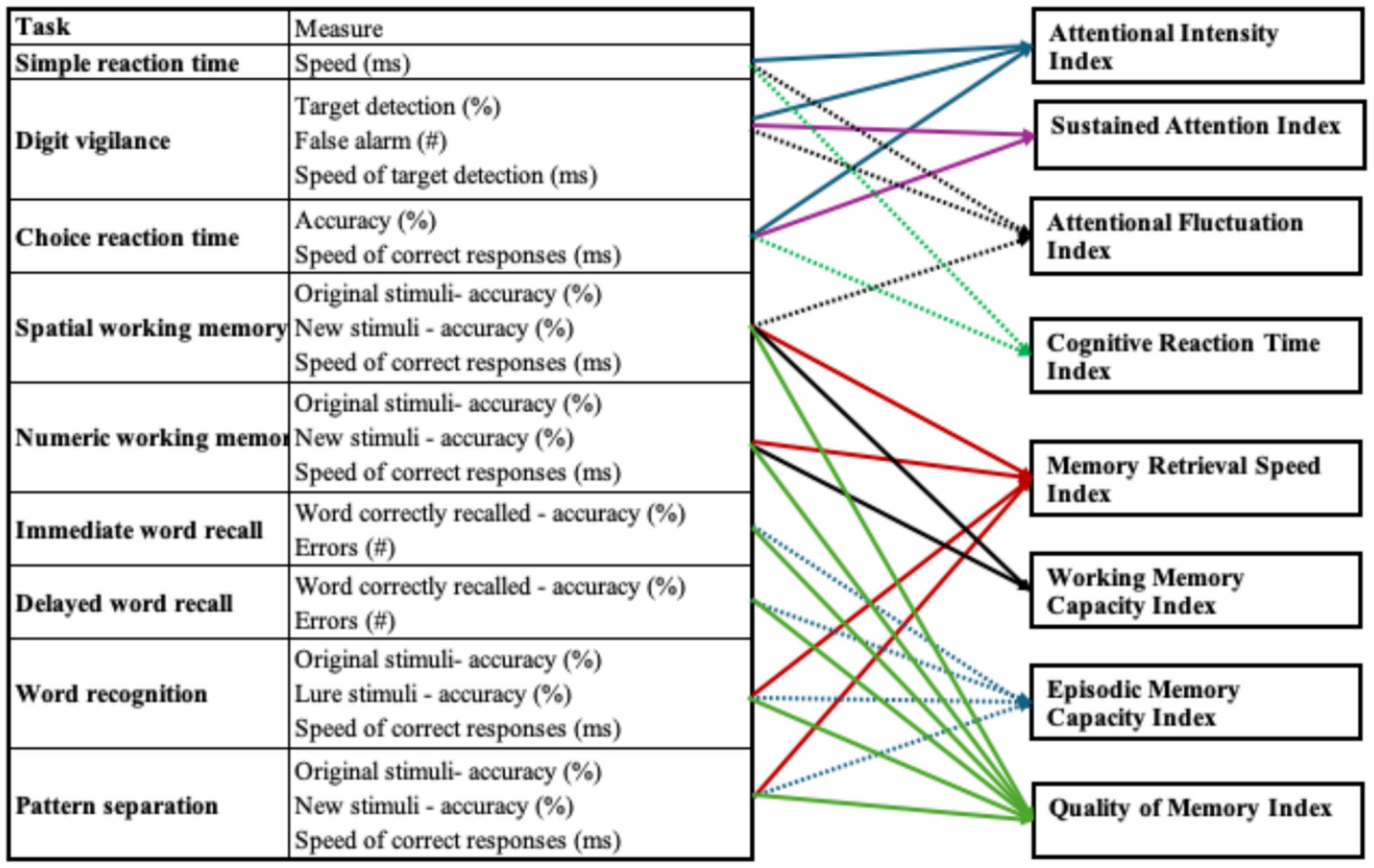
Graphic representation of the core measures for the CogTrack battery. Adapted from Wesnes et al. (41), with permission from Sage Publications. Showing (from left to right) the running order of tasks, individual task outcome measures and the composition of the eight indices derived by factor analysis. Arrows indicate that a task outcome measure contributes to the given index: Attentional intensity, Sustained attention, Attentional fluctuation, Memory retrieval speed, Cognitive reaction, Working memory, Episodic memory and Quality of Memory.

The eight indices of core measures on CogTrack combine the same task measures as used in the CDR system to form the factor scores named: attentional intensity, sustained attention, attentional fluctuation, cognitive reaction time, working memory, memory retrieval speed, episodic memory and quality of memory. Composite scores, or cognitive indices, were developed and used in interventions assessing cognitive functioning such as (42), and (31). The calculation of each index was performed as described in (30). A diagram to show the components of each index can be seen in Figure 2.

### Mood scale: bond-Lader VASs of mood and alertness

To assess mood, the Bond-Lader visual analogue mood scales (43) were employed, as they have been used in various nutritional randomised controlled trials (RCTs) (41, 44, 45). Following the recommended procedures (43), the three mood factors’ alert’, ‘calm’ and ‘content’ were formed by combining scores from the 16 Bond-Lader visual analogue scales. These scales have previously shown validity and reliability (46).

### Profile of mood scale

The Profile of Mood States (POMS) questionnaire (47) was used to monitor whether the overall mood status of the participants in the morning before administering treatment changed during the three sessions of the trial. This well-established method tool has been utilised in various nutrition interventions similar to previous work (41) and (48). The POMS is a factor-analytically derived measure of psychological distress that has demonstrated high levels of reliability and validity (49).

### Blood glucose

The blood glucose levels were measured at baseline, 45, 90 and 135 min post-supplementation using a finger prick-puncture, which utilised a portable BG monitor system (ACCU-CHEK One call-EZ, Roche Ltd., Basel, Switzerland). The ACCUCHEK reader has a reported coefficient of variance (CV) of less than 5%.

### Study visits

**Figure 3:**
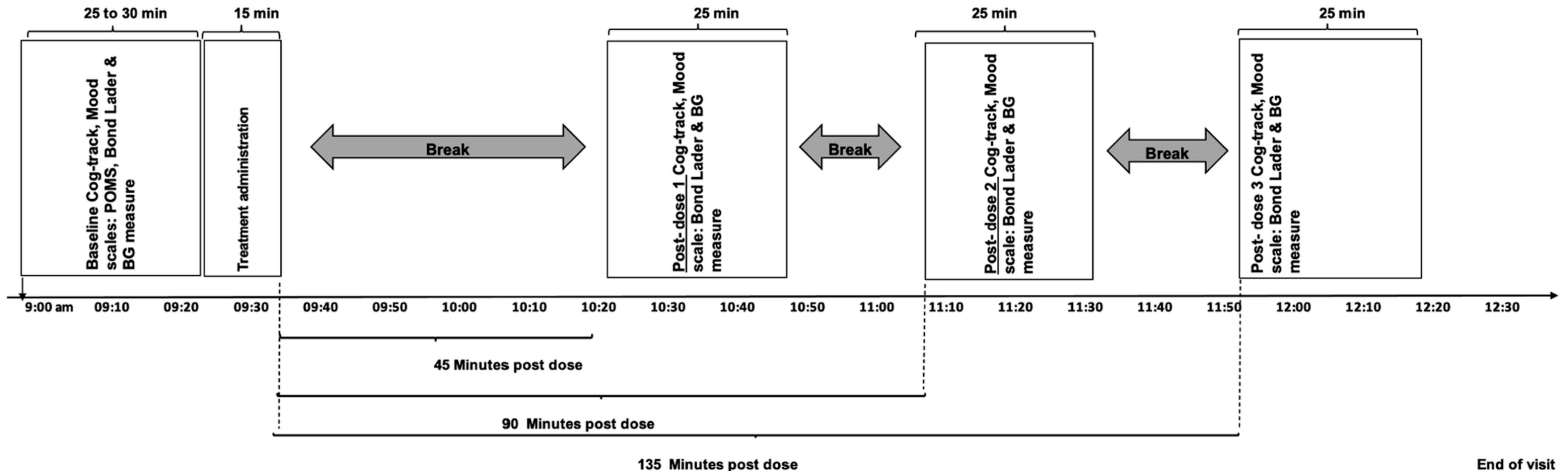
Study session timeline.

Before each study visit, participants were asked to avoid alcohol and avoid consumption of dark fruit and dark fruit juices for 24 h prior to their arrival, as well as to fast and refrain from consuming caffeine since 21:00 the previous evening (a 12-h fast). On each of the three study visits, all participants were required to arrive at the laboratory at 09:00 a.m., with each visit separated by 7 days as shown in Figure 3. See details in the Participant Information Sheet, in the Supplementary material.

The baseline assessments included CogTrack (CDR System), BG, Bond-Lader and the POMS scales. Each volunteer was then given the allocated treatment yoghurt and a cup (150 mL) of water. Participants were requested to consume all of the treatment within 15 min. Post-consumption of the treatment, assessments repeated at 45, 90- and 135 min were BG and the Bond-Lader mood scales and the CogTrack system, also shown in Figure 3. Each study visit lasted approximately 3.3 h.

### Statistical analyses

The novelty of this research posed a challenge to pre-defining a single outcome as the primary outcome due to insufficient comparable prior research to predict which cognitive domains would be sensitive to the phenolic content of dates. Therefore, a Bonferroni adjustment for multiple comparisons was implemented, as explained in (50). This decision was not mentioned in the protocol, since although the utilised cognitive tasks are statistically independent, the common practice in this field is typically not to adjust for multiple statistical comparisons. However, during the trial (before completion of data collection and analysis) the lack of consistency in outcomes among published studies using the same or similar testing batteries promoted us to adopt this more cautious approach (see details in (38)).

Linear mixed model analyses (MML) were employed on the change from baseline data for all outcomes, including CogTrack assessments and Bond-Lader mood scale outcomes. The study treatments (three levels), time of testing (three levels), and the interaction between study treatment and time of testing were included as fixed factors. The SPSS statistical package, version 24 for Windows, was used to perform the analysis. In instances where significant main effects of treatment and/or interactions were identified, pairwise comparisons were carried out using simple mixed model repeated measures (MMRM) analyses of variance (ANOVAs) with multiple comparisons.

For the Bonferroni correction, the alpha level of 0.05 was divided by the number of outcomes measured for cognitive tasks: 30 for the 30 individual cognitive tasks (resulting in *α* = 0.0017), eight for the eight measures for the CogTrack indices (*α* = 0.0063), and three for the three measures for the Bond-Lader mood scales (*α* = 0.0167).

While the *p*-value is an informative indicator of whether an effect exceeds what could be expected from random variation, Cohen’s d is a measure of effect size for comparing two means. As one of the most common methods for measuring effect size in psychology research, Cohen’s d was used to estimate the effect size of the outcomes that reached significance with the Bonferroni correction.

For POMS mood scale outcomes, baseline scores of the three study conditions were compared using MMRM ANOVAs with Greenhouse–Geisser correction to monitor whether there were any systematic variations between sessions during the trial, across the study conditions.

Additionally, all data were coded prior to analysis to maintain the blinding of study participants, investigators, and statisticians.

## Results

All study data are publicly available in Zenodo at https://zenodo.org/records/16984634/files/NU_DATES_2018-04-22_V1.03%20original.xlsx?download=1.

The number of participants dropped from 36 to 35, due to an IT malfunction causing missing data in the baseline scores, requiring exclusion of this participant’s data set from the data analysis of all measures. All figures are provided in the Supplementary material to allow full visualisation of the dataset.

### Baseline

#### Initial values for outcome measures

The actual scores of the baseline assessment of each visit for the CogTrack measures, Bond-Lader mood scale factors and the MMRM ANOVA’s methodology were compared between intervention conditions (i.e., across study visits in the crossover design). None of the analyses showed statistically significant differences (all *p* > 0.05); thus, the MML analysis proceeded as planned and shown in Table 3.

**Table 3:** The mean and standard error for pre-dose baseline and change from baseline and ANOVA for the individual tasks of CogTrack following the administration of two different varieties of date fruit (Barhi, Khassab) and a placebo mixed with 0% fat yoghurt (*n* = 35; 23 females and 12 males).

Task	Measure	Treatment	Baseline	Post-dose change			Effect of treatment		Effect of treatment*time	
			0 Min	45 min	90 min	135 min	F	P	F	P
Immediate word recall	Correct words %	Barhi	44.4 ± 19.8	−0.95 ± 13.82	−0.00 ± 16.41	0.57 ± 14.58	3.79	0.02	0.14	0.97
		Khassab	44.3 ± 14.8	0.76 ± 22.56	−2.2 ± 19.40	0.76 ± 18.49				
		Placebo	47.9 ± 21.1	−5.52 ± 12.98	−6.85 ± 12.67	−4.38 ± 13.07				
	Number of incorrect words (errors)	Barhi	0.58 ± 0.77	0.20 ± 0.68	0.06 ± 1.00	−0.03 ± 0.79	0.47	0.62	0.16	0.96
		Khassab	0.42 ± 0.69	0.14 ± 1.06	0.14 ± 1.03	0.03 ± 0.89				
		Placebo	0.56 ± 0.81	0.00 ± 0.73	0.09 ± 0.89	−0.11 ± 0.83				
Simple reaction time	Average Speed msec	Barhi	354 ± 117	1.85 ± 80.94	−6.81 ± 95.69	12.02 ± 104.88	0.85	0.43	0.08	0.99
		Khassab	349 ± 65	8.26 ± 40.87	11.58 ± 45.52	13.02 ± 49.20				
		Placebo	376 ± 164	−6.72 ± 108.57	−16.24 ± 144.11	−0.17 ± 178.00				
Digit vigilance	Average speed (msec)	Barhi	467 ± 67	3.00 ± 13.82	2.89 ± 15.95	3.79 ± 15.61	8.60	0.00103*	0.48	0.75
		Khassab	463 ± 47	−1.70 ± 7.90	−1.81 ± 10.22	−4.90 ± 12.06				
		Placebo	465 ± 50	−2.91 ± 6.47	−1.23 ± 8.13	−1.90 ± 8.52				
	Targets Detected %	Barhi	90.5 ± 15.5	0.15 ± 65.28	−4.00 ± 62.31	0.90 ± 65.34	2.94	0.05	0.61	0.66
		Khassab	92.7 ± 8.7	12.25 ± 30.46	3.01 ± 20.91	15.74 ± 33.67				
		Placebo	92.5 ± 10.0	17.43 ± 24.31	16.49 ± 36.89	6.27 ± 39.55				
	False Alarms	Barhi	3.67 ± 5.94	−1.26 ± 5.65	−1.11 ± 5.28	−1.34 ± 5.65	3.03	0.05	0.15	0.97
		Khassab	3.14 ± 3.96	−0.69 ± 1.81	−0.51 ± 1.92	−0.31 ± 2.31				
		Placebo	1.97 ± 1.93	−0.09 ± 1.66	−0.31 ± 1.92	0.34 ± 2.62				
Choice reaction time	Accuracy %	Barhi	95.89 ± 3.13	0.34 ± 3.71	0.51 ± 3.57	−0.29 ± 3.46	0.18	0.84	0.24	0.92
		Khassab	95.78 ± 3.57	0.69 ± 2.82	−0.23 ± 2.94	−0.74 ± 4.23				
		Placebo	95.17 ± 3.98	0.57 ± 4.24	−0.23 ± 4.10	−0.51 ± 4.53				
	Average Speed (msec)	Barhi	472 ± 70	11.41 ± 79.14	−0.94 ± 57.98	0.22 ± 66.10	1.24	0.29	0.03	1.00
		Khassab	462 ± 67	19.30 ± 98.51	3.30 ± 75.44	10.92 ± 73.38				
		Placebo	472 ± 78	4.66 ± 65.49	−9.15 ± 51.96	−8.63 ± 65.45				
Spatial working memory	Original Stimuli Accuracy %	Barhi	94.1 ± 11.9	−2.14 ± 9.08	−1.07 ± 6.51	−2.32 ± 7.43	1.77	0.17	0.09	0.99
		Khassab	94.8 ± 5.9	−1.96 ± 11.11	−1.96 ± 12.57	−2.14 ± 9.93				
		Placebo	93.0 ± 8.8	−1.78 ± 12.63	0.36 ± 11.13	0.89 ± 9.72				
	New Stimuli Accuracy %	Barhi	94.2 ± 106	−2.43 ± 13.63	1.00 ± 5.92	−0.86 ± 7.12	2.31	0.10	0.68	0.61
		Khassab	96.3 ± 5.5	−0.57 ± 9.98	−2.29 ± 9.87	−1.43 ± 7.03				
		Placebo	94.6 ± 8.6	1.14 ± 9.48	1.29 ± 8.94	1.29 ± 9.87				
	Original Stimuli Average Speed (msec)	Barhi	709 ± 19	−27.70 ± 206.87	−40.21 ± 140.03	−33.95 ± 161.24	0.80	0.45	0.20	0.94
		Khassab	707 ± 208	−66.12 ± 168.36	−62.49 ± 169.96	−52.38 ± 179.65				
		Placebo	653 ± 202	−15.15 ± 214.60	−29.17 ± 199.77	−52.79 ± 170.82				
	New Stimuli Average Speed (msec)	Barhi	798 ± 265	−64.41 ± 148.99	−39.34 ± 112.78	−8.62 ± 166.20	0.21	0.81	1.28	0.28
		Khassab	739 ± 209	−44.83 ± 156.31	−31.96 ± 121.21	−33.27 ± 177.39				
		Placebo	691 ± 160	2.76 ± 142.26	−28.82 ± 146.32	−51.19 ± 123.60				
	Average Speed	Barhi	761 ± 213	−48.16 ± 153.38	−39.04 ± 104.19	−18.72 ± 143.57	0.44	0.65	0.68	0.61
		Khassab	725 ± 203	−53.88 ± 150.65	−45.18 ± 127.71	−42.23 ± 168.54				
		Placebo	675 ± 170	−5.46 ± 156.83	−28.39 ± 154.67	−52.26 ± 127.27				
Numeric working memory	Original Stimuli Accuracy %	Barhi	93.9 ± 6.8	−0.19 ± 9.76	−1.14 ± 12.67	−2.85 ± 10.51	0.30	0.74	0.47	0.80
		Khassab	93.2 ± 7.4	−1.33 ± 8.21	−1.14 ± 10.53	0.38 ± 9.69				
		Placebo	93.3 ± 8.4	−1.14 ± 10.73	−2.47 ± 10.10	−1.71 ± 8.75				
	New Stimuli Accuracy %	Barhi	93.9 ± 16.8	0.19 ± 6.36	0.19 ± 6.76	0.00 ± 6.26	3.79	0.02	0.41	0.80
		Khassab	97.4 ± 4.0	−2.66 ± 7.08	−1.90 ± 7.15	−2.66 ± 7.61				
		Placebo	96.9 ± 4.6	−0.95 ± 6.69	−3.04 ± 7.29	−1.71 ± 8.13				
	Original Stimuli Average Speed (msec)	Barhi	658 ± 193	2.91 ± 95.38	−30.23 ± 1 34.79	−21.48 ± 1 58.57	1.36	0.26	0.43	0.79
		Khassab	603 ± 113	13.85 ± 74.70	22.84 ± 217.29	8.98 ± 80.11				
		Placebo	604 ± 130	−14.17 ± 103.20	−20.61 ± 80.94	9.51 ± 239.63				
	New Stimuli Accuracy (msec)	Barhi	702 ± 191	4.44 ± 154.12	−25.13 ± 174.15	52.64 ± 246.56	0.49	0.61	0.83	0.51
		Khassab	685 ± 158	−19.64 ± 149.65	−31.52 ± 114.18	16.40 ± 188.89				
		Placebo	655 ± 124	8.56 ± 118.47	1.27 ± 131.67	−13.46 ± 148.97				
	Average Speed (msec)	Barhi	689 ± 198	3.39 ± 105.98	−27.34 ± 140.19	19.06 ± 195.53	0.04	0.96	0.23	0.92
		Khassab	645 ± 123	−3.22 ± 101.60	−6.90 ± 130.33	11.85 ± 124.16				
		Placebo	630 ± 119	−1.88 ± 100.00	−9.89 ± 88.38	−2.36 ± 166.00				
Delayed word recall	Correct words %	Barhi	38.0 ± 18.9	−7.42 ± 14.07	−6.66 ± 13.52	−10.66 ± 14.11	4.27	0.01	0.62	0.65
		Khassab	34.8 ± 17.4	−7.61 ± 22.41	−10.47 ± 21.46	−5.52 ± 20.09				
		Placebo	40.6 ± 19.9	−13.52 ± 15.46	−15.61 ± 14.45	−13.90 ± 15.70				
	Number of incorrect words (errors)	Barhi	0.75 ± 1.05	0.14 ± 1.12	0.03 ± 1.01	0.03 ± 1.22	0.04	0.96	0.50	0.74
		Khassab	0.64 ± 0.80	0.09 ± 1.22	0.14 ± 1.30	0.03 ± 1.15				
		Placebo	0.72 ± 0.91	−0.06 ± 1.08	0.09 ± 1.09	0.31 ± 1.27				
Word recognition	Original Stimuli Accuracy %	Barhi	79.45 ± 13.08	−1.33 ± 12.81	3.23 ± 13.94	1.33 ± 9.80	7.33	0.0024	1.02	0.40
		Khassab	75.37 ± 13.97	−1.71 ± 14.93	1.90 ± 17.07	2.66 ± 17.20				
		Placebo	77.04 ± 12.79	−3.80 ± 11.11	−7.80 ± 13.08	−4.95 ± 16.67				
	New Stimuli Accuracy %	Barhi	88.70 ± 13.78	−5.33 ± 15.15	−3.42 ± 11.92	−8.95 ± 13.70	0.04	0.97	0.62	0.65
		Khassab	91.67 ± 9.48	−4.00 ± 9.17	−6.09 ± 12.87	−6.66 ± 12.20				
		Placebo	91.30 ± 12.73	−4.76 ± 9.91	−6.85 ± 11.02	−6.47 ± 13.95				
	Original Stimuli Average Speed (msec)	Barhi	945 ± 439	13.38 ± 728.14	−69.99 ± 489.41	−135.58 ± 452.11	2.09	0.13	0.64	0.64
		Khassab	796 ± 222	15.58 ± 350.58	66.69 ± 353.96	106.93 ± 134.61				
		Placebo	782 ± 216	24.31 ± 257.80	−25.07 ± 305.31	−31.18 ± 236.72				
	New Stimuli Average Speed (msec)	Barhi	936 ± 251	−11.72 ± 194.67	−36.58 ± 170.05	−77.78 ± 183.73	1.23	0.29	1.48	0.21
		Khassab	893 ± 342	−44.01 ± 288.84	−40.30 ± 310.30	114.09 ± 687.62				
		Placebo	812 ± 233	67.74 ± 284.72	−4.01 ± 249.52	14.27 ± 275.90				
	Average Speed (msec)	Barhi	944 ± 296	5.53 ± 390.28	−51.68 ± 263.53	−113.44 ± 266.88	1.74	0.18	1.66	0.16
		Khassab	849 ± 251	−35.90 ± 202.14	3.85 ± 257.62	106.09 ± 629.24				
		Placebo	793 ± 194	51.43 ± 239.54	−13.35 ± 182.20	−11.74 ± 184.78				
Pattern separation	Original Stimuli Accuracy %	Barhi	81.53 ± 14.78	−2.57 ± 14.47	−2.43 ± 16.42	−5.71 ± 13.94	0.36	0.70	0.28	0.89
		Khassab	76.94 ± 15.37	1.14 ± 14.95	−3.57 ± 16.20	−3.00 ± 16.27				
		Placebo	74.72 ± 17.65	−1.00 ± 12.17	−2.29 ± 16.42	−4.86 ± 14.01				
	New Stimuli Accuracy %	Barhi	79.03 ± 13.03	−6.43 ± 11.73	−3.57 ± 11.85	−6.86 ± 13.23	1.19	0.31	0.19	0.94
		Khassab	77.36 ± 13.86	−0.86 ± 13.90	−2.14 ± 15.20	−4.43 ± 16.12				
		Placebo	79.44 ± 15.01	−3.71 ± 14.77	−3.43 ± 17.48	−5.71 ± 17.62				
	Original Stimuli Average Speed (msec)	Barhi	1,049 ± 314	59.25 ± 240.41	53.16 ± 345.56	−0.86 ± 295.10	0.18	0.83	0.27	0.90
		Khassab	1,081 ± 309	69.27 ± 790.00	−21.11 ± 464.27	−8.69 ± 460.93				
		Placebo	918 ± 220	26.15 ± 182.89	69.15 ± 378.72	50.12 ± 429.89				
	New Stimuli Average Speed (msec)	Barhi	1,065 ± 184	138.67 ± 678.08	89.80 ± 424.86	−39.25 ± 173.08	1.73	0.18	0.87	0.48
		Khassab	1,125 ± 619	60.61 ± 447.42	−49.79 ± 223.30	−77.71 ± 278.57				
		Placebo	974 ± 230	39.96 ± 263.89	119.24 ± 514.37	104.53 ± 704.96				
	Average Speed (msec)	Barhi	1,057 ± 227	88.19 ± 378.03	66.09 ± 346.53	−21.50 ± 210.00	0.64	0.53	0.67	0.61
		Khassab	1,098 ± 408	70.30 ± 609.29	−34.35 ± 264.69	−28.62 ± 252.67				
		Placebo	948 ± 214	25.77 ± 201.67	77.50 ± 393.34	77.92 ± 567.43				

Unadjusted *p*-values are presented. Bonferroni-corrected significance threshold for individual cognitive tasks was *α* = 0.0017, as described in the Statistical Analysis section. * Indicates statistically significant differences between conditions (Bonferroni-corrected).

#### POMS

The same MMRM ANOVA methodology was undertaken for the six POMS mood scale factor scores (named depression, tension, fatigue, confusion and vigour, plus the overall score named total mood disturbance). During the three or more weeks that each participant was enrolled in the study, a small but significant reduction was observed (Table 4) in the tension score (*p* < 0.003). No other POMS factor showed a significant effect (all *p* > 0.05), and all trends were negative (Table 4) and no time x treatment interactions were observed.

**Table 4:** Mean ± SD values for POMS subscales and Total Mood Disturbance across the three study visits. Lower values indicate more neutral mood (less disturbance).

	***Visit***	***Mean ± SD***	***F-Value***	***P-Value***
Tension	1	11.11 ± 7.11	6.94	0.003
	2	10.29 ± 7.58		
	3	8.54 ± 6.50		
Depression	1	9.71 ± 9.54	0.39	0.68
	2	9.71 ± 10.62		
	3	8.83 ± 8.87		
Anger	1	7.57 ± 6.57	0.71	0.51
	2	6.91 ± 7.36		
	3	6.66 ± 5.98		
Vigour	1	14.6 ± 5.98	2.87	0.07
	2	13.9 ± 5.78		
	3	12.1 ± 5.84		
Fatigue	1	8.25 ± 5.63	1.48	0.83
	2	7.82 ± 5.63		
	3	6.62 ± 5.44		
Confusion	1	8.51 ± 4.53	0.44	0.65
	2	8.14 ± 5.22		
	3	7.71 ± 4.72		
TMD	1	32.2 ± 31.12	1.13	0.64
	2	29.5 ± 31.12		
	3	26.2 ± 26.71		

### Cognitive test individual task outcomes

**Figure 4:**
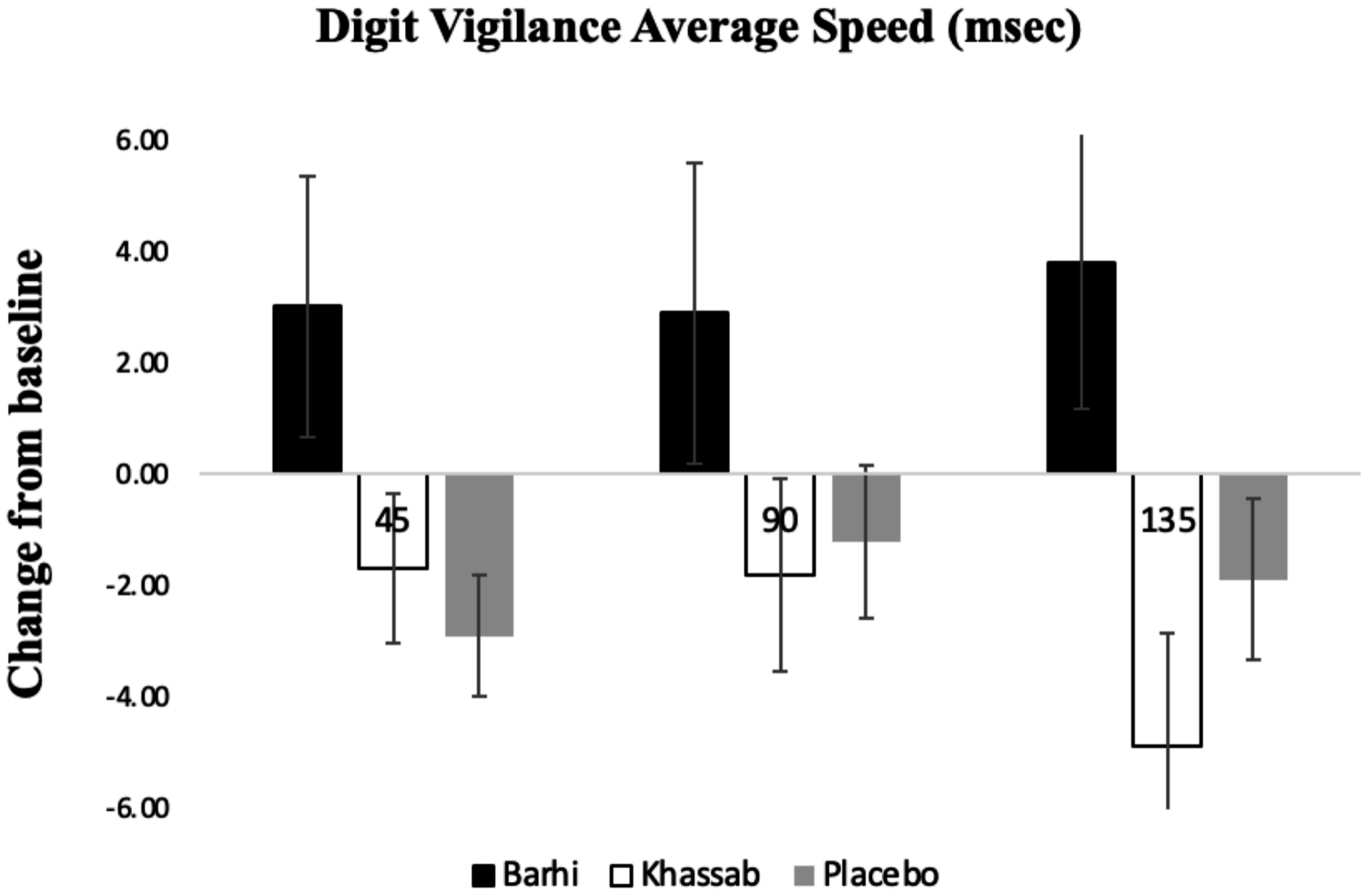
Main effects of change from baseline scores for Digit Vigilance response speed, the one CogTrack task with significant effects. Mean changes from baseline (±SE) are shown for the Barhi, Khassab, and Placebo treatments at 45, 90, and 135 min post-consumption.

All outcomes are expressed as changes from baseline, and statistical comparisons reflect differences in these changes between intervention conditions. In the digit vigilance task, a significant main effect of treatment was observed on the average response speed (milliseconds; *F* = 8.60, *p* = 0.001, Table 3). Pairwise comparisons indicated that Barhi resulted in a significantly longer reaction time compared with Khassab (*p* = 0.001), reflecting slower performance. This effect represented a significant difference in change from baseline between the two date conditions, with neither treatment differing significantly from placebo. The effect of Khassab did not statistically significantly differ from placebo (*p* = 0.87), the effect size for Barhi was medium (*d* = 0.42), see Figure 4. None of the other tasks reached a *p*-value below the *α* of 0.0017.

### Bond-Lader, indices of cognitive assessment and BG

**Figure 5:**
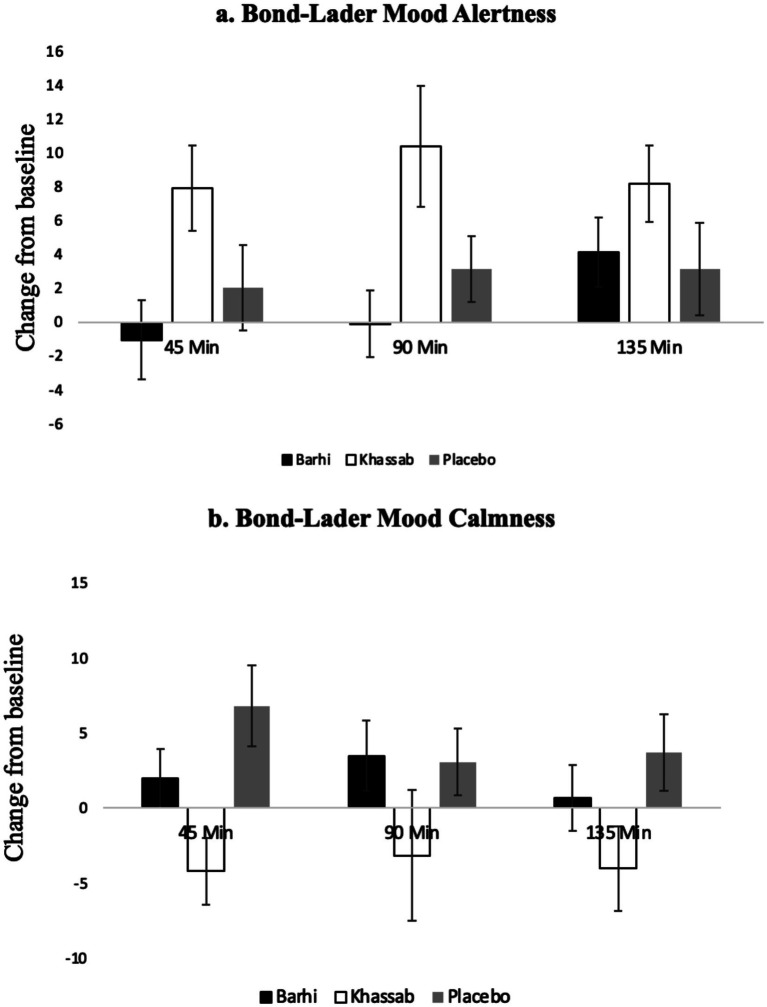
Main effects of change from baseline scores for the Bond-Lader mood indices with significant effects. **(a)** Alertness mood index, and **(b)** calmness mood index. Scores are presented as means and standard errors.

A statistically significant main effect in change from baseline between treatments (p-value below the α of 0.0167) was detected (*F* = 6.033, *p* = 0.003), and the pairwise comparison with Placebo showed an increase in the Bond-Lader alertness level (Table 5; Figure 5) that was significant for Khassab (*p* = 0.005) but not for Barhi (*p* = 0.63), with a significant difference between the two date-containing treatments (*p* = 0.001). The main effect of treatment was also significant for the Bond-Lader calmness index (Table 5; Figure 5; *F* = 4.87, p = 0.001). Pairwise comparisons indicated a significant reduction in calmness (change from baseline) compared with placebo following Khassab intake (*p* = 0.001), whereas the increase associated with Barhi was not statistically significant (*p* = 0.43). A significant difference was also found between the two date treatments (*p* = 0.02). Correspondingly, Khassab showed medium effect sizes for both the increase in alertness levels (*d = 0.61*) and the decrease in calmness levels (*d = 0.75*), see Figure 5.

**Table 5:** The mean and standard error for pre-dose baseline and change from baseline and *ANOVA* for CogTrack indices, Bond-Lader mood and BG measures following the administration of two different varieties of date fruit (Barhi, Khassab) and a placebo mixed with 0% fat yoghurt (*n* = 35; 23 females and 12 males).

Measures	Treatments	Baseline	Post-dose change			Effect of treatment		Effect of treatment* time intervals	
		0 Min	45 Min	90 Min	135 Min	F	*p*	F	*p*
Attentional intensity index	Barhi	1,230 ± 22	2.16 ± 12.15	8.22 ± 13.31	13.86 ± 15.85	0.74	0.48	0.83	0.51
	Khassab	1,223 ± 23	15.12 ± 15.12	11.28 ± 10.37	29.66 ± 13.67				
	Placebo	1,235 ± 24	28.62 ± 11.50	14.77 ± 9.23	11.69 ± 13.32				
Sustained attention index	Barhi	87.71 ± 2.22	3.24 ± 1.70	3.20 ± 1.73	3.10 ± 2.25	3.64	0.038	0.40	0.81
	Khassab	89.69 ± 1.07	0.59 ± 1.02	−0.57 ± 1.11	−2.84 ± 1.80				
	Placebo	89.47 ± 1.36	−0.79 ± 1.36	−0.50 ± 1.19	−1.85 ± 1.25				
Attentional fluctuation index	Barhi	73.47 ± 4.58	3.71 ± 6.27	−6.82 ± 4.98	1.09 ± 5.82	1.76	0.18	0.65	0.62
	Khassab	67.91 ± 4.42	7.89 ± 5.70	2.76 ± 5.55	6.21 ± 5.13				
	Placebo	76.79 ± 5.72	−3.32 ± 5.68	−2.88 ± 4.92	−3.63 ± 5.12				
Memory retrieval speed index	Barhi	3,029 ± 74	−55.68 ± 42.60	115.41 ± 52.93	−125.92 ± 54.59	2.46	0.09	0.86	0.49
	Khassab	2,918 ± 75	69.81 ± 155.97	−19.73 ± 63.79	110.62 ± 142.43				
	Placebo	2,780 ± 72	20.89 ± 35.93	28.65 ± 56.35	−30.81 ± 62.12				
Cognitive reaction time	Barhi	138.26 ± 7.09	6.18 ± 37.97	6.27 ± 41.45	9.67 ± 34.11	1.16	0.32	0.94	0.44
	Khassab	124.67 ± 7.27	12.86 ± 29.79	7.63 ± 36.93	10.75 ± 36.74				
	Placebo	130.52 ± 8.44	7.06 ± 38.75	6.42 ± 42.33	8.82 ± 38.15				
Working memory capacity index	Barhi	188.18 ± 1.50	−4.38 ± 3.62	0.12 ± 1.79	−3.18 ± 2.02	1.37	0.26	0.81	0.52
	Khassab	189.14 ± 1.75	−5.20 ± 3.95	−6.16 ± 4.56	−6.24 ± 3.07				
	Placebo	184.24 ± 3.14	0.01 ± 3.51	−1.40 ± 2.91	0.47 ± 3.09				
Episodic memory capacity index	Barhi	204.71 ± 12.04	−26.33 ± 8.98	−13.43 ± 8.18	−30.29 ± 7.54	3.134	0.05	1.83	0.13
	Khassab	194.00 ± 9.79	−13.81 ± 11.43	−24.57 ± 9.20	−16.57 ± 9.74				
	Placebo	203.38 ± 11.13	−31.95 ± 7.97	−44.00 ± 7.49	−41.62 ± 9.01				
Quality of memory index	Barhi	200.27 ± 7.48	−16.16 ± 6.48	−8.41 ± 5.98	−21.75 ± 5.44	0.16	0.85	1.94	0.11
	Khassab	198.09 ± 6.68	−10.95 ± 7.99	−22.11 ± 6.96	−15.90 ± 6.59				
	Placebo	194.90 ± 8.50	−14.61 ± 5.95	−23.15 ± 6.28	−18.87 ± 5.50				
Bond-Lader mood (Alertness)	Barhi	61.22 ± 2.49	−1.05 ± 2.34	−0.10 ± 1.98	4.14 ± 2.05	6.03	0.006*	0.93	0.45
	Khassab	55.46 ± 2.27	7.91 ± 2.53	10.38 ± 3.56	8.19 ± 2.25				
	Placebo	59.42 ± 2.79	2.04 ± 2.52	3.14 ± 1.94	3.15 ± 2.72				
Bond-Lader mood (Contentment)	Barhi	64.02 ± 3.09	1.97 ± 1.85	3.20 ± 1.60	3.66 ± 1.43	0.11	0.90	0.46	0.76
	Khassab	65.01 ± 2.56	2.05 ± 2.18	2.91 ± 4.45	2.06 ± 2.18				
	Placebo	64.23 ± 2.54	2.76 ± 1.58	4.69 ± 1.46	2.78 ± 1.76				
Bond-Lader mood (Calmness)	Barhi	58.22 ± 2.47	2.00 ± 1.95	3.49 ± 2.36	0.67 ± 2.22	4.87	0.014*	0.62	0.65
	Khassab	62.17 ± 2.61	−4.20 ± 2.22	−3.17 ± 4.37	−4.04 ± 2.80				
	Placebo	57.89 ± 2.09	6.81 ± 2.68	3.07 ± 2.23	3.69 ± 2.55				
Blood glucose concentration	Barhi	4.75 ± 0.09	1.11 ± 0.15	0.11 ± 0.10	−0.05 ± 0.08	1.40	0.25	0.55	0.70
	Khassab	4.88 ± 0.07	0.94 ± 0.22	−0.12 ± 0.20	−0.34 ± 0.18				
	Placebo	4.81 ± 0.08	0.73 ± 0.20	0.02 ± 0.14	−0.07 ± 0.12				

Unadjusted *p*-values are presented. Bonferroni-corrected significance thresholds were *α* = 0.0063 for CogTrack indices and *α* = 0.0167 for Bond–Lader mood scales, as described in the Statistical Analysis section. * Indicates statistically significant differences between conditions (Bonferroni-corrected).

No main treatment effects on change from baseline, post-treatment session effects, or interactions with *p*-value below the *α* of 0.0063 were detected for any of the eight CogTrack indices (Table 5).

**Figure 6:**
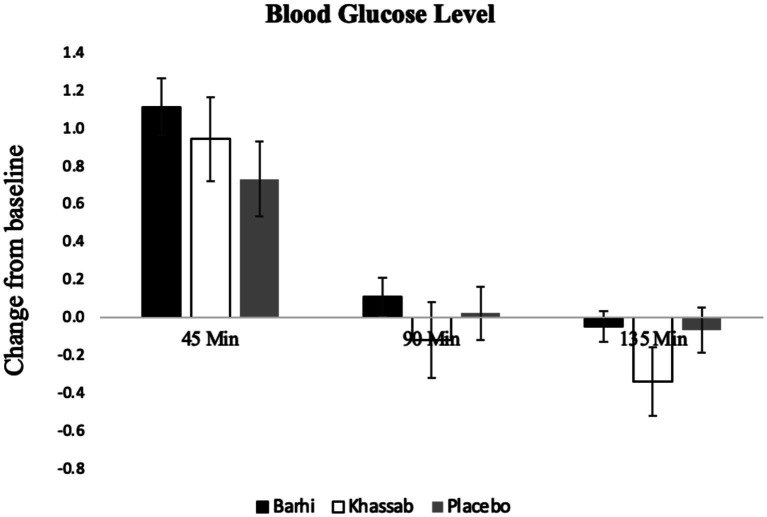
Main effects of change from baseline scores for blood glucose levels at 45, 90, and 135 min post-consumption. Error bars represent the standard error of the mean (SE).

With *p* = 0.25 for effect of treatment and *p* = 0.70 for treatment x time interaction, the BG results confirmed that the addition of sugar to the same level as treatment with the highest natural content successfully equalised both overall effect and timing of the resulting impact on plasma concentrations, see Figure 6.

Table 5 provides an overview of the overall changes from baseline for all CogTrack outcomes, Bond-Lader mood factors, and blood glucose measurements. Researchers with specific interest in one or more of these outcomes (e.g., for the purpose of systematic data collection, in which case the Bonferroni adjustment is irrelevant) are encouraged to access the trial’s full data on Zenodo and/or communicate with the corresponding author to obtain the specific results they need.

### Treatment guesses (assessment of blinding/masking effectiveness)

Out of the 36 participants, 27 correctly identified which weeks they received the placebo treatment, with 9 incorrect answers. Since the expected random distribution would be 24 correct and 12 incorrect, the Chi-square showed *p* = 0.437, confirming that the participants’ ability to identify the treatment as date or placebo was not significantly better than random chance.

## Discussion

This study demonstrated that the performance of the date-containing treatments was nearly equivalent to the placebo effect for most outcomes, except for a few outcomes where one or the other individual date treatment showed different effects compared to the other cultivar and/or the placebo. In other words, no particular pattern was observed that could be matched with any mechanistic hypothesis. Therefore, despite employing a conservative statistical approach, the observed statistically significant effects of the individual date treatments might still be a result of a Type 1 error (51). For this reason, we have not further discussed outcomes which did not retain significance after the Bonferroni adjustment, even if *p* < 0.05, nor elaborated on the time course trends shown on the figures. The purpose of publishing these results is therefore primarily to make the results of this carefully designed study available to the scientific community: to add to the general body of knowledge and as inspiration for future research (which could involve replication of some or all of the elements of this trial), rather than to announce any surprising new discovery. Although all calculated effect sizes indicate only minor to moderate changes, they are still sufficiently high to justify additional future research on Barhi and Khassab – initially to confirm whether the effects are reproducible, and if so, to investigate the active constituents and mechanisms. The POMS results reflected changes in baseline mood scores across study visits. The POMS analyses demonstrated that participants progressively became less tense during the trial, presumably reflecting increased familiarity with the testing procedures. This trend was expected and did not require any adjustment or raise concerns regarding the interpretation of the trial outcomes, particularly given the fully balanced study design.

### Potential effect of phenolic content or other constituents in enhancing cognitive functions

The significant increase in ‘alertness’ and decrease in ‘calmness’ after consumption of Khassab compared to both Barhi and placebo treatments aligns with a study (52) that utilised the same mood assessment tool to examine the effects of different doses of a special coffee blend containing 520.89 mg of chlorogenic acid. In that study, the same increase in alertness was reported in a healthy elderly population, with no accompanying changes in calmness. Moreover, consuming anthocyanin-enriched blackcurrant juice (525 ± 5 mg/60 kg) showed some trends toward increased alertness without influencing calmness in one study (32), but the opposite effect was observed for purple grape juice with a dose of 150.4 mg of anthocyanin in a study by Haskell-Ramsay et al. (44), where it increased the self-rating ‘calmness’ of young, healthy volunteers.

In contrast, Barhi was associated with a significant slowing in digit vigilance reaction time compared with Khassab, indicating reduced performance. This observed effect was not aligned with the phenolic content, as Barhi contained only 184.04 mg GAE/115 g, less than half of Khassab; it may instead reflect the influence of other unmeasured phytochemical date constituents, such as alkaloids (3). Furthermore, the supplementary analysis of glucose levels showed no consistent differences in the pattern of the participants’ blood glucose levels at the three time intervals (Figure 6).

The oral ingestion of glucose has an established enhancing effect in the literature. Among most cognitive function indices, verbal episodic memory appears to be particularly sensitive to glucose ingestion, as noted in the review by Smith et al. (53) on the ‘glucose memory facilitation effect’. Furthermore, a meta-analysis by Riby (54) of the effects of glucose on cognitive function concluded that episodic and working memory, verbal episodic, and visuospatial memory are more likely to show highly significant overall benefits of glucose in young, healthy volunteers. At the same time, sugar consumption did not have any significant effects on other indices, such as executive function, motor function, attention, semantic retrieval, and implicit memory. Moreover, the reported dose of glucose to be the optimum for improving memory was 25 g, with more minor benefits observed at higher doses, notably at 50 g. However, our study compensated for this potential confounding effect of the sugar content of the dates, by ensuring equal total sugar content of the treatments of 45.37 g, including lactose from the yoghurt, of which 16.78 g was glucose.

Date fruit has been proposed as rich in phenolic content, and in our study, 115 g of fresh date fruit flesh provided a Total Phenolic Content of 184 and 424 mg of GAE, equivalent to 1.60 and 3.69 mg of GAE/g FW for Barhi and Khassab, respectively: this is a relatively small concentration compared to the different quantities of polyphenols from other sources applied in the studies cited above (11, 12).

### Strengths

The treatments developed for the present study successfully masked the presence of the date fruit flesh (since the guesses on which week was the placebo were not significantly different from random chance).

The study design ensured that any effects were not due to the sugar or fibre content.

The design was intended to assess dose dependency of the content of phenolic acids in the dates, if any of the outcomes had shown such an association (which was not the case).

While only a few outcomes retained significance after Bonferroni adjustment, all the measured outcomes may be relevant in other contexts, such as inclusion in meta-analyses.

### Limitations

The literature available before the trial did not provide sufficient background information to select a single primary outcome, Therefore, it was necessary to treat the measured multiple outcomes as equivalent within each category of outcomes, using Bonferroni correction with *α* values corresponding to the number of outcomes in each category.

Since the study design focused on testing effects of phenolic acids, the results did not provide any information about other potentially relevant date fruit flesh constituents.

## Conclusion

The current study results found that even when applying a Bonferroni adjustment, the administration of the Khassab date cultivar enhanced alertness and reduced calmness, while Barhi slowed down the Digit Vigilance response, in a “young and healthy” adult cohort. These effects were not consistently linked with content of phenolics nor with any other measured constituents of the dates. Future studies of dates should take into account the possibility of other active constituents in the fruit flesh.

## Data Availability

The original contributions presented in the study are included in the article/Supplementary material, further inquiries can be directed to the corresponding author/s.
