# Supplementary material for: Acute effects of fruit flesh of two Saudi date varieties differing in phenolic content and compared with a sugar- and fibre-matched placebo on mood and cognition in healthy young volunteers: a randomised, double-blind, crossover study
Source: Front Nutr. 2026 Jul 22;13:1733218. doi: 10.3389/fnut.2026.1733218 (PMC13440079; doi:10.3389/fnut.2026.1733218)
Supplement: Supplementary file 1 [file Table_1.DOCX]

**Supplementary figures**

Digit Vigilance Average Speed (msec)

Change from baseline

|  | Barhi | Khassab | Placebo |
| --- | --- | --- | --- |
| 45 | 3 | -1.7 | -2.91 |
| 90 | 2.89 | -1.81 | -1.23 |
| 135 | 3.79 | -4.9 | -1.9 |

**Figure S 1** Main effects of change from baseline scores for Digit Vigilance response speed, the one CogTrack task with significant effects. Mean changes from baseline (±SE) are shown for the Barhi, Khassab, and Placebo treatments at 45, 90, and 135 minutes post-consumption.

**Figure S 2** Main effects of change from baseline scores for the Bond-Lader mood indices with significant effects.

(a) alertness mood index, and (b) calmness mood index. Scores are presented as means and standard errors.

**Figure S 3** Main effects of change from baseline scores for blood glucose levels at 45, 90, and 135 minutes post-consumption. Error bars represent the standard error of the mean (SE).
